# Anxiety and Depression in Adults With Congenital Heart Disease

**DOI:** 10.3389/fped.2022.906385

**Published:** 2022-06-21

**Authors:** Corinna Lebherz, Michael Frick, Jens Panse, Philipp Wienstroer, Katrin Brehmer, Gunter Kerst, Nikolaus Marx, Klaus Mathiak, Hedwig Hövels-Gürich

**Affiliations:** ^1^Department of Cardiology, University Hospital RWTH Aachen, Aachen, Germany; ^2^Department of Oncology, Hematology, Hemostaseology and Stem Cell Transplantation, University Hospital RWTH Aachen, Aachen, Germany; ^3^Center for Integrated Oncology Aachen Bonn Cologne Düesseldorf (CIO-ABCD), Aachen, Germany; ^4^Department of Cardiology, DIAK Hospital, Schwäbisch Hall, Germany; ^5^Department of Pediatric Cardiology, University Hospital RWTH Aachen, Aachen, Germany; ^6^Department of Psychiatry, Psychotherapy and Psychosomatics, University Hospital RWTH Aachen, Aachen, Germany

**Keywords:** anxiety, depression, ACHD adult congenital heart disease, locus of control, Hospital Anxiety and Depression Scale (HADS)

## Abstract

**Introduction:**

Anxiety and depression can worsen outcome in patients with heart disease. We elucidate the prevalence of anxiety and depression in a cohort of adults with congenital heart disease (ACHD).

**Materials and Methods:**

Prospective screening for anxiety or depression was performed in 204 consecutive patients of the outpatient clinic of our tertiary care center using the Hospital Anxiety and Depression Scale (HADS) questionnaire and the distress thermometer (DT) as a potential ultra-short screening test. Functional data were assessed at liberty of the responsible physician. HADS scores ≥ 8 were considered doubtful and scores ≥ 11 as confirmed cases of anxiety or depression, respectively. HADS results were compared with a historical group of 100 patients with non-Hodgkin Lymphoma (NHL) as well as German reference values from the literature.

**Results:**

Patients from the ACHD cohort were 28 ± 10 years old (mean ± *SD*, 54% male), 34% had a simple, 51% a moderate, including 52 patients with transposition of the great arteries after arterial switch operation, and 15% a heart defect of severe complexity. Prevalence of depression in ACHD was comparable to the German normal population (5.9% ACHD vs. 5.4% control). In contrast, prevalence of anxiety was higher than expected from reference values (12.7% ACHD vs. 5.6% control). There was a positive association between psychological distress and NYHA class [anxiety: OR 2.67 (95% CI, 1.50–4.76) *p* = 0.001; depression: OR 2.93 (95% CI, 1.60–5.35) *p* = 0.0005], but not with age, gender, or heart defect severity. Percentages of patients with ACHD with anxiety were significantly higher than in a cohort of patients with indolent non-Hodgkin lymphoma (NHL) but comparable to those with aggressive NHL (HADS-A ≥ 11: ACHD 12.7%, indolent NHL 2.2%, aggressive NHL 13.2%; *p* = 0.037 ACHD vs. indolent NHL; *p* = 0.929 ACHD vs. aggressive NHL). The distress thermometer screening test had only a fair discriminatory ability (AUC 0.708; *p* = 0.002) and is therefore of limited usability.

**Conclusion:**

Adults with congenital heart disease exhibit an increased risk for anxiety disorders independently of the severity of the underlying heart defect. Anxiety prevalence was comparable to a historical cohort of patients with aggressive NHL underlining the importance of a routine screening for psychosocial distress in adults with congenital heart disease.

## Introduction

Most of the children born with congenital heart defects survive well into adulthood. However, depending on the complexity of the heart defect, the children might need to undergo several staged operations during their first years of life. In patients with very complex heart disease, even then, only a palliative situation can be accomplished. However, life-long cardiac supervision is mandatory not only for patients with severe congenital heart defects, but also with mild or moderate congenital heart defects to detect potential problems, arising from not yet cared for lesions or late sequelae of previous operations, early enough to circumvent long-lasting limitations of the cardiac function.

Previous studies evaluated anxiety and depression levels as well as the quality of life in children and adults with congenital heart disease. Quality of life was mainly affected by subjective measures such as functional NYHA class and socio-demographic factors (1, 2) whereas objective measures such as the severity of the underlying heart defect or maximum exercise capacity did not. Indeed, due to lifestyle adaptations and resilience adults with congenital heart disease tend to overestimate their functional capacity (3). Besides the quality of life, the prevalence of depression and anxiety is significantly increased in patients with congenital heart disease afflicting up to 30% of the study population (4, 5). The long-term cardiovascular effects of this phenomenon are not yet determined.

The link between psychological distress and worse cardiovascular outcome has been well established in patients with acquired heart disease as depression is a known independent predictor of all-cause mortality in patients with acquired heart failure (6). As such, the risk for major cardiovascular events in the coronary heart disease is increased 3.7 times in patients with anxiety disorder and 3.1 times in patients with depression (7). In addition, patients with coronary artery disease and concomitant depression or anxiety have increased all-cause mortality (8). Vice versa, psychosocial stress is a risk factor for the development of myocardial infarction (9).

Therefore, besides the physical wellbeing of their patients, cardiologists should also spotlight concealed psychosocial distress as it has been emphasized by a recent position statement of the European Association of Preventive Cardiology (10). Questionnaires such as the “Hospital Anxiety and Depression Scale (HADS)” or structured interviews are time consuming, and hence difficult to incorporate into clinical routine. Thus, easily implementable screening tests are needed for a wide acceptance of patients and doctors.

The aim of this study was to evaluate prevalence of anxiety and depression in adults with congenital heart defects presenting in the out-patient clinic of our specialized tertiary care center. In addition, we wanted to assess the sensitivity of the ultra-short-scale pencil and paper “distress thermometer (DT)” as a potential screening tool being frequently applied in psycho-oncology settings (11).

## Materials and Methods

This is a single center prospective cohort study which was conducted between 01 October 2015 and 31 December 2016. During a routine visit in the outpatient clinic of the specialized tertiary care for adults with patients with congenital heart defect were asked to complete four questionnaires to detect psychosocial distress and collect demographic data. Participation was optional, meaning that the routine follow-up was performed independently of the study. Inclusion criteria were age ≥ 18 years, presence of congenital heart disease and ability to consent. Clinical examination and cardiac diagnostic tests were performed at liberty of the responsible physician independent of the study. Complexity of the heart defect was classified as simple (class I), moderate (class II), or severe (class III) according to the Warnes classification. The study was approved by the ethics committee CTC-A Nr. 14-159. The following questionnaires were used:

(1) Distress thermometer (DT): an ultra-short screening tool consisting of a visual analog scale drawn as a thermometer (12). The scale ranges from 0 (no distress) to 10 (severe distress). The patient has to mark his subjective stress level during the past week. There is a complementary list with various problem areas that can be checked as well.

(2) Hospital Anxiety and Depression Scale (HADS): a self-rating questionnaire developed for patients with somatic diseases, consisting out of 14 questions/items. The score for each item can range between 0 and 3. The questionnaire covers 7 items for depression and 7 items for anxiety. Scores for each question are added up. A score ≥ 8 is considered as suspect and a score ≥ 11 as probable for the respective condition (13). Results were compared with published reference values (14) and a historical control of 100 patients with indolent or aggressive NHL published previously. (15).

(3) Questionnaire for the locus of control (LoC): analyses to what extent the individual believes in the fact that health and disease can be impacted internally by the person itself or externally by caregivers or just by fate (16). The test encompasses 21 questions using a Likert-type style.

### Statistics

Statistical analysis has been performed using SPSS IBM statistics software version 28.

Baseline characteristics and test scores were compared between the groups using two-sample *t*-tests for continuous variables and Pearson chi-square tests for categorical data.

Multivariable ordinal logistic regression analyses were performed with HADS anxiety or depression levels as a dependent variable. The variables Warnes classification and NYHA classification are ordinal parameters. Gender, history of arrhythmia, use of cardiovascular drugs, and reduced ejection fraction of the main chamber are binary parameters. Age was the only metric parameter.

Binary logistic regression analysis evaluating the association of HADS questionnaire with Locus of Control or Distress Thermometer performed with HADS anxiety or depression levels as dependent variable. HADS–A (anxiety) or HADS–D (depression) levels ≥ 8 were coded as 1. The three Locus of Control subscales are included as ordinal denominator.

Receiver operating characteristic curve (ROC) analyses were performed according to the SPSS ROC curve tool with HADS–A or HADS–D levels ≥ 8 or ≥ 11 as binary parameter.

A *p*-value less than 5% was considered as statistically significant in each analysis.

## Results

### Study Population Characteristics

In total, 204 patients completed the questionnaires along with their routine cardiology tests, reflecting approximately 25% of all patients with ACHD consulting the outpatient clinic during the study period. Most of the participants had a congenital heart defect of moderate complexity (51%, *n* = 103) including a large group of patients with transposition of the great arteries after arterial switch operation (*n* = 52). One third of patients had a congenital heart defect of simple complexity (34%, *n* = 70), 15% of severe complexity (*n* = 31) (Table 1).

**TABLE 1:** Overview of the distribution of the underlying heart defects.

	Heart defect	*n*	%
I- mild	Aortic valve disease	34	16.7
	Mitral valve disease	6	2.9
	Congenital AV-block III	1	0.5
	Cor triatrium sinistrum	1	0.5
	ASD II	9	4.4
	ASD I or sinus venosus defect	5	2.5
	VSD	14	6.9
II- moderate	Coarctation aorta	14	6.9
	AVSD (partial, complete)	7	3.4
	Pulmonary valve disease	5	2,5
	Fallot tetralogy	23	11.2
	Tricuspid valve disease	2	1.0
	dTGA-arterial switch	52	25.5
III- severe	dTGA-Senning/Mustard	9	4.4
	ccTGA	2	1
	Truncus arteriosus	3	1.5
	Pulmonary atresia	7	3.5
	Univentricular heart	10	4.9

Baseline characteristics of the evaluated patient cohort are depicted in Table 2. In total, 54% of the participants were male (*n* = 111), 46% female (*n* = 93) with a mean age of 28 ± 10 years. More than half of the patients underwent at least one operation in the past (57% one operation, 14% two, 13% more than two operations). In the majority of cases, echocardiographic systolic function of the systemic ventricle was normal (80%, *n* = 163) and only about one-third of the patients had a cardiovascular medication (35%, *n* = 71). In total, 27% of the participants had a history of arrhythmia and 7% a cardiac device implanted. Following the clinical criteria, 63% of the patients were in NYHA class I (no symptoms or limitation during exercise, *n* = 128), 33% in NYHA class II (strenuous exercise causes symptoms, *n* = 67), and only 4% in NYHA class III (marked limitation of exercise capacity, less than ordinary activity causes symptoms, *n* = 8). This went along with a self-reported exercise frequency exceeding once a week in 45% of the patients (*n* = 92), once a week in 24% of the patients (*n* = 49), once a month in 9% (*n* = 19), and less than once a month or never in 6% (*n* = 12) and 14% of the patients (*n* = 29), respectively.

**TABLE 2:** Cardiovascular baseline characteristics of the study population.

	Baseline characteristics		Severity congenital heart defect		
				
		All *n* (%)	Mild *n* (%)	Moderate *n* (%)	Severe *n* (%)
		204	70	104	30
**Age**	Mean ± *SD* (min-max)	28 ± 10 (18–59)	29 ± 11 (18–59)	26 ± 9 (18–57)	30 ± 9 (18–54)

**Gender**	Male	111 (54)	36 (51.4)	58 (55.8)	17 (56.7)
	Female	93 (46)	34 (48.6)	46 (44.2)	13 (43.3)

**Number of cardiac operation**	0	30 (15)	26 (37.1)	3 (2.9)	1 (3.3)
	1	117 (57)	33 (47.1)	76 (73.1)	8 (26.7)
	2	28 (14)	6 (8.6)	18 (17.3)	4 (13.3)
	≥ 3	26 (13)	4 (5.7)	7 (6.8)	15 (50)

**NYHA class**	I	128 (63)	47 (67.1)	73 (70.2)	8 (26.7)
	II	67 (33)	20 (28.6)	30 (28.8)	17 (56.7)
	III	8 (4)	2 (2.9)	1 (1)	5 (16.7)

**Exercise**	Never	29 (14)	7 (10)	12 (11.5)	10 (33.3)
	<1 a month	12 (6)	6 (8.6)	3 (2.9)	3 (10)
	Once a month	19 (9)	6 (8.6)	10 (9.6)	3 (10)
	Once a week	49 (24)	17 (24.3)	25 (24)	7 (23.3)
	Several times a week	92 (45)	32 (45.7)	53 (51)	7 (23.3)

**Pacemaker/ICD**		14 (7)	4 (5.7)	2 (1.9)	8 (26.7)

**History of arrhythmia**		55 (27)	15 (21.4)	20 (19.2)	20 (66.7)

**Cardiac medication**	Yes	70 (34)	27 (38.6)	20 (19.2)	23 (76.7)
	Beta-blocker	39 (19)	16 (22.9)	10 (9.6)	13 (43.3)
	ACE-inhibitor, ARB	21 (10)	11 (15.7)	5 (4.8)	5 (16.5)
	Diuretics	8 (4)	2 (2.9)	2 (1.9)	4 (13.3)
	MRA	4 (2)	1 (1.4)	0	2 (6.5)
	Antiarrhythmic drugs	5 (2.5)	1 (1.0)	0	4 (13.3)
	OAK	34 (17)	16 (22.9)	4 (3.8)	14 (46.7)
	ASS	9 (4.4)	2 (2.9)	1 (1)	6 (20)

**Systemic ventricle systolic function**	Normal	163 (80)	59 (84.3)	94 (90.4)	10 (33.3)
	Mildly reduced	14 (6,8)	3 (4.3)	5 (4.8)	6 (20)
	Moderately reduced	3 (1,5)	0	1 (1)	2 (6.7)
	Severely reduced	2 (1)	0	1 (1)	1 (3.3)

**Cardiovascular risk factors**	Arterial hypertension	20 (10)	8 (11.4)	10 (9.6)	2 (6.7)
	Diabetes mellitus	3 (1.5)	2 (2.9)	0	1 (3.3)
	Smoking	26 (13)	8 (11.4)	12 (11.5)	6 (20)
	Hypercholesterolemia	14 (7)	6 (8.6)	4 (3.4)	4 (13.3)

**Non-cardiac comorbidities (all)**		83 (40.7)	33 (47.1)	36 (34.6)	14 (46.7)
Neurologic disorder		22 (10.8)	9 (12.9)	12 (11.5)	1 (3.3)
Psychiatric disorder		25 (12.3)	8 (11.4)	12 (11.5)	5 (16.7)

Cardiovascular risk factors were dominated by smoking (13%, *n* = 26), arterial hypertension (10%, *n* = 20), and dyslipidemia (7%, *n* = 14) whereas diabetes was only reported rarely (1.5%, *n* = 3). In total, 12% of the participants had a history of a psychiatric disease and 6% took a psychiatric medication.

### Prevalence of Anxiety Disorder

Anxiety and depression levels were evaluated using the HADS questionnaire. The items of the questionnaire can be sorted into the HADS-A (anxiety) and HADS-D (depression) subscale to distinguish both conditions. Scores equal or above 8 in each subscale suggest the presence of anxiety or depression, whereas scores equal or above 11 affirm the diagnosis with a high certainty.

**FIGURE 1:**
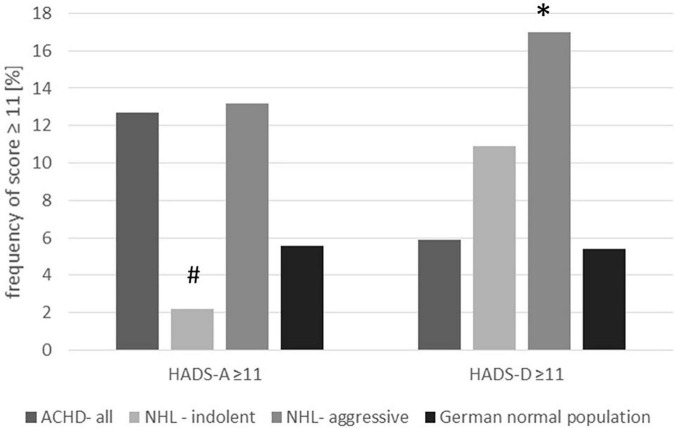
Percentage of subjects with confirmed cases of anxiety or depression (HADS cut-off value ≥ 11). The figure compares the congenital heart disease cohort with the lymphoma cohort and the reference value from the literature. *p = 0.009 ACHD vs. aggressive lymphoma; ^#^*p* = 0.037 ACHD vs. indolent lymphoma.

There were signs for an anxiety disorder with a HADS-A score ≥ 8 points in 22.5% and evidence for anxiety with a HADS–A score ≥ 11 in 12.7% of the participants of the whole congenital heart disease patient cohort of our study. According to Hinz and Brahler (14), percentages of subjects with HADS–A cut-off values ≥ 8 and ≥ 11 in the German population were 21% and 6.8% (*n* = 4,410, 55% women, age 50.3 ± 17.2 years), respectively. In those subjects with an age less than 40, more closely resembling our ACHD study group, the percentages of participants scoring ≥ 8 and ≥ 11 in the HADS–A anxiety subscale were 17.4% and 5.6%, respectively (*n* = 609 men and *n* = 833 women). Just looking at confirmatory cases with an HADS–A score ≥ 11, the prevalence of anxiety in the ACHD group is higher than expected if compared to the published reference value (12.7% ACHD vs. 5.6% control). Since a statistical comparison with the results of the literature was not possible, we included a historical cohort of patients with indolent or aggressive non-Hodgkin lymphoma (NHL) investigated previously in the Department of Psychiatry, Psychotherapy, and Psychosomatics of our hospital. This study group comprises of 100 patients with NHL (44 women, 61.3 ± 13.6 years). For detailed baseline characteristics, we refer to the original analysis (15). There was no difference in percentages of subjects meeting the criteria HADS–A ≥ 8 between patients with congenital heart disease and indolent or aggressive lymphoma (HADS–A ≥ 8: ACHD 22.5%, NHL indolent 21.7%, NHL aggressive 34%; ACHD vs. NHL indolent: *p* = 0.90; ACHD vs. NHL aggressive *p* = 0.25) Table 3. The number of cases with strong signs for anxiety reflected by an HADS–A ≥ 11 was significantly higher in the ACHD group compared with those patients with indolent NHL but comparable to those patients with aggressive NHL (HADS–A ≥ 11: ACHD 12.7%, NHL indolent 2.2%, NHL aggressive 13.2%; ACHD vs. NHL indolent *p* = 0.037; ACHD vs. NHL aggressive *p* = 0.929) (Figure 1).

**TABLE 3:** Percentage of subjects reaching the HADS cut-off values ≥ 8 or ≥ 11 in the respective groups and HADS as well as DT mean + *SD* values if available.

	HADS-A mean (*SD*)	HADS-A ≥ 8 *n* (%)	HADS-A ≥ 11 *n* (%)	HADS-D mean (*SD*)	HADS-D ≥ 8 *n* (%)	HADS-D ≥ 11 *n* (%)	DT mean (*SD*)
**Study group- ACHD**							
All	5.20 (4.19)	46 (22.5)	26 (12.7)	3.07 (3.49)	22 (10.8)	12 (5.9)	4.7 (2.5)
Simple complexity	6.03 (4.69)	22 (31.4)	14 (20.0)	3.80 (4.15)	11 (15.7)	7 (10.0)	4.7 (2.3)
Moderate complexity	4.52 (3.53)	16 (15.4)	8 (7.7)	2.52 (2.89)	7 (6.7)	3 (2.9)	4.7 (2.5)
Severe complexity	5.60 (4.83)	8 (26.7)	4 (13.3)	3.27 (3.48)	4 (13.3)	2 (6.70)	5.0 (2.6)
TGA (ASO)	4.06 (3.62)	7 (13.5)	4 (7.7)	2.44 (2.76)	3 (5.8)	0 (0)	5.0 (2.6)
**Historical control–NHL**							
All	5.23 (3.86)	28 (28.3)	8 (8.1)	5.14 (4.38)	26 (26.3)	14 (14.1)	
Indolent	4.46 (3.51)	10 (21.7)	1 (2.2)	4.57 (4.37)	9 (19.6)	5 (10.9)	
Aggressive	5.91 (4.16)	18 (34.0)	7 (13.2)	5.64 (4.36)	17 (32.1)	9 (17.0)	
**Normative reference according to Hinz and Brahler (14)**		(21.0)	(6.8)		(23.7)	(9.4)	

### Prevalence of Depression

Hospital Anxiety and Depression Scale sub-scale Depression score ≥ 8 was found in 10.8%, HADS–D score ≥ 11 in 5.9% of the patients with congenital heart disease. The number of patients with depression was therefore comparable to published HADS data of the German reference population (14) (HADS–D ≥ 11: 5.9% ACHD vs. 5.4% control group age less than 40) as well as the reported prevalence of major depressive disorder in the German population according to Jacobi using structured interviews [(17) overall 12 month prevalence of major depressive disorder 6%, age group 18–34 years 9%, age group 35–49 6.5%; see Table 3]. Percentage of subjects with an HADS–D score ≥ 11 or HADS–D score ≥ 8 were significantly lower in patients with congenital heart disease compared with patients with aggressive lymphoma (HADS–D ≥ 8: ACHD 10.8%, aggressive NHL 32.1%, ACHD vs. aggressive NHL: *p* = 0.001; HADS–D ≥ 11: ACHD 5.9%, aggressive NHL 17%; ACHD vs. aggressive NHL, *p* = 0.009), but comparable to patients with indolent lymphoma (HADS–D ≥ 8; indolent NHL 19.6%, ACHD vs. indolent NHL, *p* = 0.103; HADS–D ≥ 11: indolent lymphoma 10.9%; *p* = 0.225 ACHD vs. indolent NHL) (see Figure 1). Previously, Westhoff-Bleck suggested a lower HADS–D cut-off value > 5 for the detection of depression in adults with congenital heart disease (18). In the current cohort, 18.6% of the participants had an HADS–D score > 5, that was significantly lower compared with the NHL patients (*p* < 0.001 vs. aggressive lymphoma, *p* = 0.027 vs. indolent lymphoma) and also less than the 25.7% reported in the study by Westhoff-Bleck (Supplementary Table 1).

### Factors Associated With Anxiety or Depression

Hospital Anxiety and Depression Scale mean values and percentage of subjects reaching the cut-off values are depicted in Table 3. There was a trend toward a lower HADS anxiety score in the congenital heart disease of moderate complexity compared with simple or severe complexity which, however, did not reach statistical significance (HADS–A mean ± *SD*: simple Warnes I 6.03 ± 4.69, moderate Warnes II 4.52 ± 3.53, severe complexity ACHD Warnes III 5.60 ± 4.83, *p* = 0.056).

Participation in the study was optional and all patients presenting in the out-patient clinic during the respective time frame were asked to participate. In the subgroup of patients with congenital heart disease of moderate complexity, we identified 52 patients with transposition of the great arteries after arterial switch operation (ASO). HADS anxiety score ≥ 8 was found in 13.5% and ≥ 11 in 7.7% of the patients with ASO which was not significantly different compared with the total group of patients with ACHD with moderate complexity or the whole ACHD cohort. However, mean HADS–A score was significantly lower in patients with ASO compared with all patients with ACHD (mean ± SD 4.06 ± 3.6 *p* = 0.183 vs. Warnes II, *p* = 0.023 vs. all ACHD; HADS ≥ 8: *p* = 0.587 vs. Warnes II, *p* = 0.069 vs. all ACHD, HADS ≥ 11: *p* = 1.00 vs. Warnes II, *p* = 0.208 vs. all ACHD) Table 3.

In the total ACHD group, there was no difference in HADS depression scores with respect to the underlying complexity of the congenital heart disease (HADS–D mean ± *SD*: simple complexity 3.8 ± 4.15, moderate complexity II 2.52 ± 2.89, severe complexity ACHD 3.27 ± 3.48, *p* = 0.056).

In the subgroup of patients with ASO, HADS depression score ≥ 8 was found in 5.8% and ≥ 11 in none. Therefore, the percentage of patients with ASO reaching the cut-off ≥ 11 was significantly lower in comparison to the whole ACHD group. However, if mean HADS–D scores were evaluated they were comparable between ASO and all patients with ACHD (ASO mean ± *SD* 2.44 ± 2.76 *p* = 0.788 vs. Warnes II and *p* = 0.134 vs. all ACHD; HADS ≥ 8: *p* = 0.696 vs. Warnes II and *p* = 0.177 vs. all ACHD, HADS ≥ 11: *p* = 0.079 vs. Warnes II and *p* = 0.037 vs. all ACHD) (Table 3).

**FIGURE 2:**
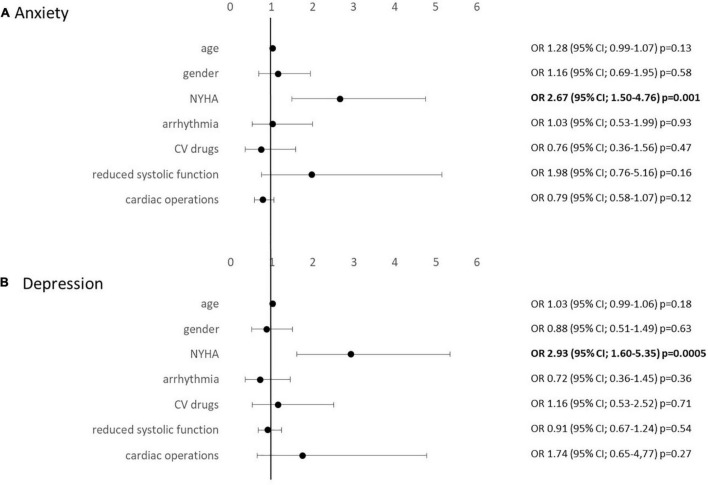
Multivariate regression analysis evaluating associations with HADS anxiety **(A)** and depression **(B)** levels.

Multivariate logistic regression analyses were performed revealing a strong association of NYHA class with levels of anxiety and depression [anxiety OR 2.67 (95% CI, 1.50–4.76) *p* = 0.001; depression OR 2.93 (95% CI, 1.60–5.35) *p* = 0.0005]. There was no correlation with age, gender, number of conducted operations, arrhythmia, CV drugs, or systolic heart function (Figure 2).

Patients with high anxiety and depression scores reported a higher frequency of doctor visits compared to those patients with normal HADS levels (anxiety *p* = 0.047; depression *p* < 0.001). In 50% of the patients with elevated anxiety scores, a psychiatric diagnosis had been made already before participation in this study, whereas in 50%, there was no previously known anxiety disorder.

In 19 patients, HADS subscales anxiety and depression were both elevated. Patients in this sub-cohort were 36 ± 12 years old (19–57 years). Warnes and NYHA class distribution (I/II/III) were *n* = 10/5/4 and *n* = 5/12/2, respectively. We did not find markers to predict both anxiety and depression.

There was no association between cardiovascular risk factors such as arterial hypertension or non-cardiac, i.e., neurological comorbidities with anxiety or depression. However, a significant positive association between pre-diagnosed psychological disorders and fulfilled HADS criteria for anxiety and depression could be confirmed (Supplementary Table 2).

### Association of Anxiety and Depression With Locus of Control and Distress Thermometer

**FIGURE 3:**
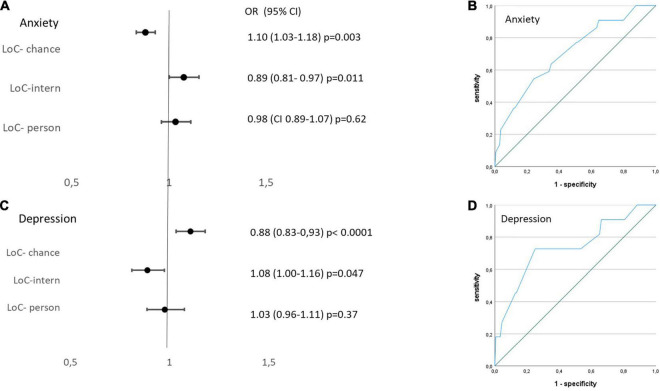
Left: Multivariate regression analysis evaluating associations of locus of control (LoC) with **(A)** HADS A-anxiety and **(C)** HADS-D-depression levels. Right: Receiver operating curve analysis evaluating discriminatory ability of Distress Thermometer for **(B)** HADS A-anxiety and **(D)** HADS-D-depression levels.

The locus of control (LoC) questionnaire evaluates the patient’s conception of its influence on disease progression with the three sub-categories chance, internal or external control. In our study, the LoC identified different personality traits of patients with high anxiety scores in contrast to those with high depression subscale levels. Patients with signs for anxiety, according to the HADS analysis, showed a significant inverse correlation with the LoC chance subscale as well as a positive association with the internal control subscale reflecting a perception of having internal control over the outcome of their life. In contrast, patients with depression showed a highly significant positive association with the LoC subscale chance and an inverse correlation with internal control indicating a personality that accredits its wellbeing mainly to chance but not to its own action (Figures 3A,C). In 19 patients presented with a combination of anxiety and depression, the feelings were dominated by a positive correlation with internal control and a negative association with chance LoC (internal control OR 1.41; CI, 95% 1.03–1.26, *p* = 0.009; chance OR 0.9; CI, 95% 0.84–0.97, *p* = 0.004).

Neither patients with anxiety nor with depression showed a correlation with the subscale external control implying that external factors such as the peer group or the responsible physician do not have an important influence on the mental state of health in this cohort.

The Distress Thermometer (DT) score revealed a significant but only fair discriminative ability to detect clinically highly relevant anxiety defined as HADS–A level ≥ 11 (AUC (area under the curve) 0.708; *p* = 0.002). A DT level > 3.25 went along with a good sensitivity of 90% but a low specificity of 36%, whereas a DT level > 4.75 detected anxiety with a sensitivity of 77% and a specificity of 47% (Figure 3B).

Comparably, clinically significant depression with HADS–D levels ≥ 11 was determined only moderately well with the DT questionnaire (AUC 0.708; *p* = 0.002). A DT level > 3.25 went along with a good sensitivity of 91% but a low specificity of 35%, whereas a DT level > 4.75 detected depression with a sensitivity of 77% and a specificity of 49% (Figure 3D). The DT was not able to reliably detect HADS–A or HADS–D levels ≥ 8 in ROC analyses (HADS–A: AUC 0.656; *p* = 0.002; HADS–D: AUC 0.688, *p* = 0.007).

## Discussion

This study shows an increased prevalence of anxiety in adult patients with congenital heart disease compared with the published reference values of the German population. Best predictor for the presence of an anxiety disorder was the functional capacity reported as NYHA class. Neither the severity of the underlying congenital heart defect nor the global systolic heart function affected the level of anxiety.

We were able to show that anxiety levels in adults with congenital heart disease are equal to patients with a malignant disorder sending a strong signal for the necessity to implement screening tools to detect psychological distress during routine cardiology follow-up examination.

The influence of personality traits and coping strategies on psychological distress has been evaluated using the LoC questionnaire. Patients who met the HADS criteria for anxiety showed high internal LoC but a low LoC subscale “chance.” This would suggest that patients with anxiety disorder rather implement coping strategies that are based on their own actions and not on external random circumstances or persons. The strong internal belief of control however might paradoxically cause distress itself (anything is the patient’s fault) and may rather yield in a worse behavioral health outcome and increased anxiety level among others reflected by an increase number of clinical appointments.

In contrast, depression levels were not increased in ACHD as we would have expected from the literature. Those patients identified with depression showed personality traits consistent with a low belief in internal control but a high conviction of the factor “chance” in their clinical wellbeing.

Kovacs was able to identify a high prevalence of lifetime mood or anxiety disorder in 50% of 280 patients with congenital heart disease, that was mainly predicted by non-disease specific factors such as loneliness and fear of negative evaluation (19). The group of Westhoff-Bleck performed a study including 150 adults with congenital heart disease, 28% of the participants showed signs of anxiety and 31% of mood/depressive disorders (20). In this study, psychiatric diagnosis was based on structural interviews as well as the HADS and the Beck Depression Inventory-2. Comparable to our results, the presence of mental disorders was negatively correlated with the functional NYHA class. Another study from the same group including 206 patients with adult congenital heart disease reported a prevalence of 25.7% for major depressive disorders. In contrast to our study, the cut-off value for the HADS–D scale was chosen much lower with a cut-off of > 5, due to an internal validation of the lower cut-off using standardized interviews. Even if the lower HADS cut-off points would be applied to our results or the mean values considered, prevalence of depression is lower than expected. The observed differences might be due to differences in the baseline characteristics or confounding co-morbidities of the respective study population. Participants of the study by Westhoff-Bleck were older and had a higher grade of congenital heart defect of severe complexity (18).

In line with the results of our study, prevalence of depression in a cohort including 767 patients with ACHD was lower than what would have been expected for the normal population (8.6%) with no relevant difference between the diagnostic heart disease groups. However, presence of depression was associated with a significant reduction of all dimensions of quality of life. In addition, depression was a stronger predictor for a decreased quality of life than a reduction in exercise capacity (21).

Psychological distress such as anxiety and depression has also been associated with non-adherence to medication and treatment in 451 outpatients with congenital heart disease. Assuming that non-adherence is associated with an increase in cardiovascular events, there is imminent need to reflect the psychological problems in cardiology follow-up visits (22). Indeed, there was an increase in cardiology clinic visits and hospitalization as well as an increased mortality observed in patients with ACHD with anxiety and depression [HR 1.40 (95% CI, 1.17–1.67 for study period depression/anxiety diagnosis, *n* = 8,334 (23)]. Vice versa, interventions focused on training the patient’s comprehension of the disease and the prognosis proved efficient in patients with acquired heart disease after myocardial infarction. Prevalence of anxiety and depression decreased 3 months post myocardial infarction in the trained group compared with the control group (24).

Kasmi et al. evaluated the prevalence of mood disorder specifically in the young adult patients with transposition of the great arteries and status post arterial switch operation (ASO) (25). The lifetime prevalence for anxiety and depressive disorders was 54 and 43%, respectively, reflecting a highly significant increase compared with the control group. In contrast, the same group reported no increase of depression or anxiety in adult patients with TGA after ASO if the current and not the life-time prevalence was evaluated for example using the HADS questionnaire (25, 26). These findings underline the need for repetitive testing for psychiatric distress in adults with congenital heart disease.

Reliable screening tools are required to implement the evaluation of the psychosocial disorders into routine cardiology follow-up visits. The ultra-short Distress Thermometer (DT) test has been validated in patients with oncological diseases and is now widely used in the clinical setting. DT levels ≥ 3 corresponded well with the HADS test result ≥ 8 with a sensitivity of 77–88% and specificity of 72–79% in 1,323 adult cancer survivors at 6 months post diagnosis in Australia (27). A Swedish group also reported a positive association of the DT with the HADS score with an area under the curve of 0.86 (95% CI, 0.82–0.9). DT score ≥ 4 was associated with a sensitivity of 87%, specificity of 73%, and negative predictive value of 95% (28).

Notably, in our cohort of patients with congenital heart disease, the correlation of the Distress Thermometer with the HADS questionnaire did not reach the acceptable sensitivity and specificity of ≥ 85 and ≥ 75%, respectively. Studies with the detailed psychometric analysis (15) and a recent meta-analysis (29) also indicated limitations to the validity of the DT.

Therefore, the evaluation of psychosocial distress in ACHD should make use of standardized questionnaires such as HADS, especially, if staff or time resources are limited and structured clinical diagnostic interviews are difficult to schedule.

## Study Limitation

Limitation of the study is the lack of structured psychiatric interviews in addition to the HADS questionnaire and also an age-matched internal control. However, validity of the HADS test has been confirmed previously in various patient groups with somatic diseases and also in the general population and may surpass some unstructured assessments (30). The updated S3 guideline in psycho-oncology even requires validated screening tools such as HADS to be conducted and not being replaced by clinical interviews. Our focus was therefore to establish a screening system also if the resources for structured clinical interviews are not available. This, of course, should not stop the physician treating to perform a clinical evaluation independently. In addition, a selection bias cannot be ruled out since participation was voluntary and only a fraction of the patients seen in the outpatient clinic did participate.

## Conclusion

This prospective evaluation of psychological disorders in a cohort of adults with congenital heart defects shows an increased prevalence of anxiety disorder in this patient group comparable to patients with an active malignant disease. Since a negative impact of anxiety on the long-term outcome of the cardiovascular disease has been suggested in various studies for acquired heart disease, cardiologists should reflect on tools to identify mood disorders and counsel the patients accordingly. Coping strategies that take the disease burden from the patient by providing external psycho-social care should be preferred.

## Data Availability Statement

The original contributions presented in the study are included in the article/Supplementary Material, further inquiries can be directed to the corresponding authors. Original data will be made available upon request within the terms of a data use agreement and within the general rules of the general data protection and ethics guidelines.

## Ethics Statement

The studies involving human participants were reviewed and approved by Ethics Committee of the RWTH Aachen. The patients/participants provided their written informed consent to participate in this study.

## Author Contributions

CL, HH-G, KB, and KM designed the study. PW, HH-G, JP, and CL acquired the data. CL, MF, and KM performed the data analysis. CL, HH-G, KM, MF, JP, GK, and NM did the composition of the manuscript. All authors contributed to the article and approved the submitted version.

## Conflict of Interest

The authors declare that the research was conducted in the absence of any commercial or financial relationships that could be construed as a potential conflict of interest.

## Publisher’s Note

All claims expressed in this article are solely those of the authors and do not necessarily represent those of their affiliated organizations, or those of the publisher, the editors and the reviewers. Any product that may be evaluated in this article, or claim that may be made by its manufacturer, is not guaranteed or endorsed by the publisher.
