# Supplementary material for: Anxiety and Depression in Adults With Congenital Heart Disease
Source: Front Pediatr. 2022 Jun 21;10:906385. doi: 10.3389/fped.2022.906385 (PMC9253420; doi:10.3389/fped.2022.906385)
Supplement: Supplementary file 1 [file Table_1.pdf]

	HADS-A mean (SD)	HADS-A > 5 n (%)	HADS-A ≥8 n (%)	HADS-A ≥11 n (%)	HADS-D mean (SD)	HADS-D > 5 n (%)	HADS-D ≥8 n (%)	HADS-D ≥11 n (%)
study group- ACHD								
all	5.20 (4.19)	76 (37.3)	46 (22.5)	26 (12.7)	3.07 (3.49)	38 (18.6)	22 (10.8)	12 (5.9)
simple complexity	6.03 (4.69)	31 (44.3)	22 (31.4)	14 (20.0)	3.80 (4.15)	16 (22.9)	11 (15.7)	7 (10.0)
moderate complexity	4.52 (3.53)	32 (30.8)	16 (15.4)	8 (7.7)	2.52 (2.89)	15 (14.4)	7 (6.7)	3 (2.9)
severe complexity	5.60 (4.83)	13 (43.3)	8 (26.7)	4 (13.3)	3.27 (3.48)	7 (23.3)	4 (13.3)	2 (6.70)
TGA (ASO)	4.06 (3.62)	13 (25)	7 (13.5)	4 (7.7)	2.44 (2.76)	8 (15.4)	3 (5.8)	0 (0)
historical control - NHL								
all	5.23 (3.86)	45 (45,5)	28 (28.3)	8 (8.1)	5.14 (4.38)	40 (40.4)	26 (26.3)	14 (14.1)
indolent	4.46 (3.51)	17 (37.0)	10 (21.7)	1 (2.2)	4.57 (4.37)	16 (34.8)	9 (19.6)	5 (10.9)
aggressive	5.91 (4.16)	18 (52.8)	18 (34.0)	7 (13.2)	5.64 (4.36)	24 (45.3)	17 (32.1)	9 (17.0)
Normative reference according to[1]								
All participants			(21.0)	(6.8)			(23.7)	(9.4)
Age <40 years			(17.4)	(5.6)			(13.7)	(5.4)

Table 1 Percentages of participants meeting the criteria for anxiety or depression using the HADS questionnaires. The cut-off points have been chosen according to the original literature (≥8 borderline, ≥11 firm) as well as according to Westhoff-Bleck ([2] recommended cut off >5 for subscale depression)

1. Hinz, A. and E. Brahler, *Normative values for the hospital anxiety and depression scale (HADS) in the general German population.* Journal of psychosomatic research, 2011. **71**(2): p. 74-8.
2. Westhoff-Bleck, M., et al., *Diagnostic evaluation of the hospital depression scale (HADS) and the Beck depression inventory II (BDI-II) in adults with congenital heart disease using a structured clinical interview: Impact of depression severity.* European journal of preventive cardiology, 2020. **27**(4): p. 381-390.

(a)

Parameter	B	Std. Error	Interval		Hypothesis Test			Exp(B)	Interval for Exp(B)	
			Lower	Upper	Wald Chi-Square	df	Sig.		Lower	Upper
arterial hypertension	0,902	0,4962	-0,071	1,874	3,303	1	0,069	2,464	0,932	6,517
diabetes	-0,319	1,3339	-2,933	2,295	0,057	1	0,811	0,727	0,053	9,926
hyperlipidemia	0,322	0,6446	-0,942	1,585	0,249	1	0,618	1,379	0,390	4,879
non-cardiac comorbidities	0,134	0,2975	-0,449	0,717	0,202	1	0,653	1,143	0,638	2,048
neurologic diseases	-0,050	0,4571	-0,945	0,846	0,012	1	0,914	0,952	0,388	2,331
psychiatric disorders	2,139	0,4592	1,239	3,039	21,707	1	**0,0000032**	8,494	3,454	20,893
(Scale)	1[a]									
Dependent Variable: HADS anxiety										

(b)

Parameter	B	Std. Error	Interval		Hypothesis Test			Exp(B)	Interval for Exp(B)	
			Lower	Upper	Wald Chi-Square	df	Sig.		Lower	Upper
arterial hypertension	0,860	0,4711	-0,064	1,783	3,329	1	0,068	2,362	0,938	5,948
diabetes	1,392	1,1680	-0,898	3,681	1,420	1	0,233	4,021	0,408	39,677
hyperlipidemia	0,110	0,5951	-1,056	1,276	0,034	1	0,853	1,116	0,348	3,583
non-cardiac comorbidities	0,420	0,3023	-0,172	1,013	1,932	1	0,165	1,522	0,842	2,753
neurologic diseases	0,414	0,4940	-0,554	1,382	0,702	1	0,402	1,513	0,574	3,983
psychiatric disorders	1,650	0,4902	0,689	2,610	11,323	1	**0,001**	5,205	1,991	13,604
(Scale)	1[a]									
Dependent Variable: HADS depression										

Table 2: Multivariate analysis evaluating the association of comorbidities on anxiety (a) or depression (b). $p < 0.05$ was considered statistically significant. Values of the respective HADS subscales were used as dependent variable.
